# Revisiting the “Christmas Holiday Effect” in the Southern Hemisphere

**DOI:** 10.1161/JAHA.116.005098

**Published:** 2016-12-22

**Authors:** Josh Knight, Chris Schilling, Adrian Barnett, Rod Jackson, Phillip Clarke

**Affiliations:** ^1^ Centre for Health PolicyMelbourne School of Population and Global HealthThe University of MelbourneVictoriaAustralia; ^2^ Section of Epidemiology & BiostatisticsSchool of Population HealthThe University of AucklandNew Zealand; ^3^ School of Public Health and Social WorkQueensland University of TechnologyBrisbaneQueenslandAustralia

**Keywords:** Christmas effect, holiday effect, seasonal mortality, seasonal variation, seasons, Epidemiology, Risk Factors, Vascular Disease, Mortality/Survival, Lifestyle

## Abstract

**Background:**

A “Christmas holiday effect” showing elevated cardiovascular mortality over the Christmas holidays (December 25 to January 7) was demonstrated previously in study from the United States. To separate the effect of seasonality from any holiday effect, a matching analysis was conducted for New Zealand, where the Christmas holiday period falls within the summer season.

**Methods and Results:**

New Zealand mortality data for a 25‐year period (1988–2013) was analyzed based on the same methodology used in the previous study. Locally weighted smoothing was used to calculate an “expected” number of deaths for each day of the year. The expected value was compared with the actual number of deaths. In addition, mean age at death was estimated and used to assess the life‐years lost due to excess mortality. There were 738 409 deaths (197 109 coded as cardiac deaths) during the period. We found evidence of a Christmas holiday effect in our of medical facility's cardiac deaths, with an excess event rate of 4.2% (95% CI 0.7–7.7%) leading to ≈4 additional deaths per annum. The average age of those with fatal cardiac deaths was 76.8 years (SD 13.5) during the Christmas holiday period, resulting in 148 to 222 years of life lost per annum.

**Conclusions:**

Cardiac mortality is elevated during the Christmas holiday period relative to surrounding time periods. Our findings are consistent with a previously reported study conducted in the United States, suggesting that cardiac mortality does not take a “summer break.”

## Introduction

A “Christmas holiday effect” on mortality has been established in the United States, with spikes in deaths from natural causes at both Christmas and New Year's Day.^1^ In the United States, however, the Christmas holiday period coincides with the coldest period of the year, when mortality rates are already seasonally high because of low temperatures and influenza.^2^ Previous studies used statistical techniques to help disentangle the holiday effect from the winter effect and found that deaths from natural causes were almost 5% higher than would be expected if the holidays did not affect mortality.^1^

Various possible explanations exist for a mortality holiday effect, including the emotional stress associated with the holidays, changes in diet and alcohol consumption,^3^ less staff at medical facilities, and changes in the physical environment (eg, visiting relatives). Nevertheless, few attempts have been made to replicate this study. A regional analysis conducted in the north of England failed to find any elevated mortality at Christmas but found a spike on New Year's Day.^4^ Although the original study indicated^1^ that future research should “seek to disaggregate the effects of winter and the winter holiday,” we are unaware any comparable studies conducted in the Southern Hemisphere, where Christmas occurs during summer, when death rates are usually at a seasonal low. In this paper, we have replicated the earlier Northern Hemisphere analysis using data from New Zealand, which allows us to separate any winter effect from the holiday effect.

## Methods

### Data

Individual‐level daily mortality data for 26 years between 1988 and 2013 were sourced from the New Zealand Ministry of Health. Access to the mortality collection data was permitted via an addendum to the data access agreement between the VIEW/PREDICT project^5^ and the New Zealand Ministry of Health. The deaths were categorized into cardiac and noncardiac groups, as defined by Phillips et al,^1^ on the basis of the International Classification of Diseases (ICD) code assigned as the primary cause of death for that person. Specifically, ICD‐9 codes 390–398, 402, 404, and 410–429 and ICD‐10 codes I00–I09, I11, I13, and I20–I51 were considered cardiac events. To allow for further decomposition of the data, the events were further categorized as to whether or not the death occurred in a medical facility. This was determined at the time of mortality coding using the location of death recorded on the death certificate. Where the recorded location of death was not a recognized medical facility, the code 9990 was recorded, indicating that the person had died outside of a medical facility.^6^ Deaths on the additional day in leap years (February 29) were removed. This meant all days in the calendar year were identically coded. To prevent any disclosure of confidential information, only data at the aggregated level were reported. This aggregation included national‐level reporting and the use of pooled ICD codes, as specified, rather than ICD codes for specific conditions.

Mortality records are centrally coded by the New Zealand Ministry of Health on the basis of death certificates completed by individual certifying physicians. The centralization of the coding is expected to reduce variability that might otherwise occur if the coding were undertaken at a facility or regional level. The possibility of misclassification remains during coding of the cause of death but is unlikely to be specific to the Christmas period because the coding is not done strictly contemporaneously. A World Health Organization report classified New Zealand as a “low ill‐defined coding” country for the coding of ischemic heart disease deaths, giving confidence that the mortality data are robust and accurate.^7^

All unique identifiers for data used in the analysis were encrypted, and the PREDICT study was approved by the Northern Region Ethics Committee Y in 2003 (AKY/03/12/314), with subsequent annual approval by the National Multiregion Ethics Committee since 2007 (MEC07/19/EXP).

We followed a previously published analysis^1^ in using a polynomial regression locally weighted smoothing (LOESS) function for daily mortality data. The bandwidth used for the LOESS was 0.11 based on the description from Phillips and colleagues of 6‐week bandwidth within an annual cycle, giving the following calculation: (6×7)/365.25. As explained by Phillips and colleagues, this method corrects for trends and seasonality but makes minimal distributional assumptions about the data. This method calculates an expected number of deaths that was compared with the actual number of deaths to detect short‐term fluctuations from an expected seasonal pattern such as a Christmas holiday effect.

The Christmas holiday period was defined as December 25 to January 7. To minimize the established impact of seasonality on mortality, the 2 weeks before (December 11–24) and after (January 8–21) the Christmas period were used as comparison periods in addition to an “all non‐Christmas” time period. The all non‐Christmas period was defined as all dates not between December 25 and January 7 and so includes the pre‐ and post‐Christmas windows.

An excess event percentage was calculated based on the difference in number of events between the actual and expected values for each day of the year, for which the expected value was defined by the LOESS data using the following formula: ((actual−expected)/expected)×100. To get a summary estimate for the impact of the Christmas holiday period on excess mortality, the mean value for all excess event percentages within the time periods (14 days for the pre‐Christmas, Christmas, and post‐Christmas values and the 351 days of the all non‐Christmas period) were calculated.

Mean ages at death and standard deviations were calculated for all mortality, all cardiac mortality, and cardiac mortality outside of a medical facility for the 4 time periods identified. To compare the age distributions of the mortality data, a *t* test was conducted and Cohen's d was calculated. Cohen's d is a measure of the size of the difference between 2 values for continuous data.^8^ It is often used in cases for which a very large sample size means that a *t* test will typically result in a statistically significant *P* value; however, this *P* value provides no information about the practical significance of the difference between the results.

To observe the impact across the age range, a cumulative distribution plot was created. An estimate of decreased life‐years was calculated on the basis of the difference in mean age at death for the Christmas period and the comparison time period, either the pre‐Christmas or all non‐Christmas period, multiplied by the number of events within the Christmas period. This value was then compared with census data to calculate an effect per million persons for the most recent time period, with the denominator provided by the 2013 New Zealand census population^9^ estimated as the “usually resident population.” The usually resident population figure was used to avoid issues with undercounting found in the raw census count figures.

As a sensitivity analysis, the Christmas holiday period was moved incrementally forward and backward from the baseline Christmas start date of December 25 to test for any boundaries of the holiday effect. To maintain comparability between results, the 2‐week time period was maintained by moving the end date in an identical manner as the start date. Because the LOESS method partially incorporates any Christmas effect in the smoothing algorithm, a sensitivity analysis was undertaken to estimate the size of this impact. This was done by calculating an expected value for each day of the Christmas period by linear interpolation using the last day of the pre‐Christmas period and the first day of the post‐Christmas period.

To detect any broad‐scale change in magnitude in the size of any Christmas effect, the 26 years of data were divided in to 4 approximately equal time periods, and subgroup analysis was undertaken, as described.

## Results

**Figure 1:**
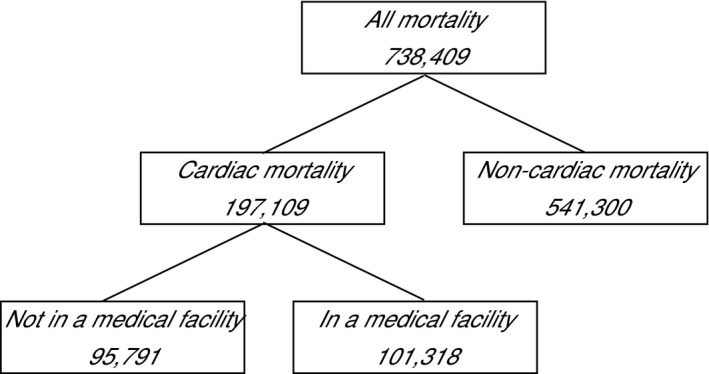
Counts of deaths between locations and cause of death.

There were 738 409 deaths (of which 197 109 where coded as cardiac deaths) in New Zealand between 1988 and 2013 (Figure 1). The average age of cardiac mortality was 76.2 years (SD 13.7) during the Christmas period compared with 77.1 years (SD 13.2) at other times of the year (Table 1). Comparing the Christmas period with the 2 weeks immediately prior to Christmas indicated that the average age of cardiac death was ≈6 months less (76.2 years [SD 13.7] versus 76.8 years [13.5], *P*=0.013) (Table 1).

**Table 1:** Age of Death for All, All Cardiac, and Cardiac Mortality Not in a Medical Facility Event Types for the Christmas, Pre‐Christmas, and Non‐Christmas Periods

Type and Location of Event	Time Period	Number of Deaths	Age at Death		
			Mean (SD), y	*t* Test	Cohen's d
All	Christmas	26 257	70.5 (21.0)	—	—
	Non‐Christmas	710 313	70.4 (21.0)	<0.001^a^	0.02
	Pre‐Christmas	26 480	70.8 (21.1)	0.16	−0.07
All cardiac mortality	Christmas	6796	76.2 (13.7)	—	—
	Non‐Christmas	189 827	77.1 (13.2)	<0.001^a^	−0.25
	Pre‐Christmas	6836	76.8 (13.5)	0.013^b^	−0.16
Cardiac mortality not in a medical facility	Christmas	3484	72.8 (14.4)	—	—
	Non‐Christmas	92 074	74.0 (13.9)	<0.001^a^	−0.32
	Pre‐Christmas	3304	73.4 (14.5)	0.15	−0.16

a *P*≤0.001.

b *P*≤0.05.

**Figure 2:**
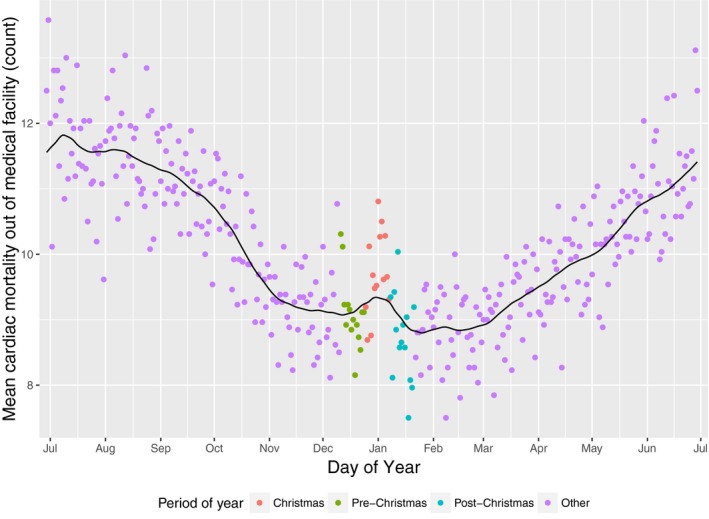
Mean number of cardiac mortality events occurring outside of a health facility between 1988 and 2013 (points) and locally weighted smoothing representing the expected values. Each point represents a mean number of mortality events for a particular calendar day, with the color coding representing the time periods of significance with regard to the impact of the holiday effect.

The mortality data had an observable Christmas holiday effect for those persons who died out of hospital from an event for which the principal code was a cardiac‐related diagnosis (Figure 2). Each point on Figure 2 represents the mean number of events by calendar date based on the 26 years of available records. The chart was centered on the Christmas period to make observations of the critical time period easier. There were 4.2% (95% CI 0.7–7.7%) more recorded events than would be expected based on the annual trends absent a Christmas effect (Table 2). There was no significant change from the seasonal trend for cardiac death within a medical facility (−0.7%, 95% CI −4.2% to 2.8%) or for overall mortality.

**Table 2:** Percentage Difference Between Calculated Mean Mortality Count and Expected Mortality Count by Type and Timing of Event

	Cardiac Deaths		Noncardiac Deaths		All Deaths	
	Not in a Medical Facility	In a Medical Facility	Not in a Medical Facility	In a Medical Facility	Not in a Medical Facility	In a Medical Facility
Pre‐Christmas	−0.77 (−4.25 to 2.71)	0.219 (−4.19 to 4.63)	−0.531 (−3.14 to 2.08)	1.134 (−0.33 to 2.59)	−0.607 (−1.99 to 0.77)	0.844 (−0.45 to 2.14)
Christmas	4.198 (0.7–7.7)^a^	−0.692 (−4.15 to 2.76)	1.779 (−1.43 to 4.99)	−0.492 (−2.77 to 1.79)	2.711 (−0.38 to 5.8)	−0.704 (−2.76 to 1.35)
Post‐Christmas	−3.173 (−6.72 to 0.37)	−1.398 (−4.19 to 1.4)	0.112 (−1.65 to 1.87)	0.546 (−1.43 to 2.52)	−0.621 (−2.49 to 1.24)	0.192 (−1.39 to 1.77)
All non‐Christmas	−0.172 (−0.8 to 0.45)	−0.058 (−0.7 to 0.59)	0.177 (−0.34 to 0.7)	0.068 (−0.28 to 0.41)	0.002 (−0.4 to 0.41)	0.047 (−0.26 to 0.36)

a *P*≤0.05.

During the 2012–2013 Christmas season, the latest for which we have full data, there were 247 cardiac deaths of which 93 were outside of a hospital facility. We estimated ≈4 additional cardiac‐related deaths out of hospital due to the Christmas holiday effect. Statistics New Zealand reported the population as 4 442 100 based on the 2013 New Zealand census.^9^ This indicates an increased event rate of ≈0.9 death per million people within the 2 weeks of the Christmas period. Based on the decreased mean age of death, between 148 and 222 life‐years (based on the pre‐Christmas and full‐year estimates, respectively) were lost due to the Christmas holiday effect.

The size of the effect was sensitive to the timing of the start of the holiday period (Figures S1 and S2), with the effect peaking in the 2 weeks starting December 27, for which a 4.5% (95% CI 1.1–7.9%) excess event rate was detected (Table S1). When the start date for a modified Christmas period was on or before December 23, there was no statistically significant Christmas effect. Likewise, if the start date was moved to or beyond December 29, no holiday effect was detected, placing upper and lower bounds on the timing of the Christmas effect. When the a linear interpolation was used to derive the expected values for the Christmas period, an increase in the effect was observed, suggesting that the LOESS method potentially underestimated the size of the effect by ≈0.8% (Figure S3, Table S2).

When a subgroup analysis of the magnitude of the Christmas holiday effect was undertaken, there was no statistically significant difference in the size of the effect between the earlier and later time periods (Figure S4). There was a nonsignificant trend toward an increasing Christmas holiday effect in the later time periods, suggesting against any attenuation of the size of the Christmas effect (Table S3).

## Discussion

We found that 4.2% more persons die from a cardiac‐related cause outside of a hospital during the Christmas period than would be expected based on long‐term seasonal trends. This is consistent with reports from the United States that also indicated an increased mortality rate during the Christmas holiday period in the Northern Hemisphere. Because the seasonality, and associated temperature profile, is opposite to the previously reported results from the Northern Hemisphere, this result supports the conclusions of Phillips and colleagues^1^ and, prior to that, Kloner et al,^3^ who indicated that the Christmas holiday effect is not associated with temperature, specifically with the cold temperatures associated with winter.

Persons who die from a cardiac event, in or out of hospital, during the Christmas period are younger by almost a year than those who die from the same or similar condition during the remainder of the year. The decrease in age at death is reduced but is still present when the comparison periods are restricted to the Christmas period and the 2 weeks prior, for which it was found that those who had a fatal cardiac event during the Christmas period were ≈6 months younger than those who died during the previous 2 weeks.

Phillips and colleagues^1^ suggested 8 further potential explanations, some of which can potentially be clarified using the New Zealand data; others remain ambiguous.

Causes for the Christmas holiday effect that are unlikely considering the New Zealand data include:

1. Respiratory diseases: Given that Christmas falls in the summer season within the Southern Hemisphere, respiratory illness seems to be an unlikely contributor to the increased mortality rate. Respiratory disease rates show a strong correlation with the winter season in New Zealand^10^ and other Southern Hemisphere countries^11^ and so are unlikely to play a major role in any Christmas holiday effect in New Zealand.
2. Increased particulate pollution: Phillips and colleagues^1^ conjectured that the increased particulate matter during their winter season might be a cause of the increased cardiac mortality rate. Pollution levels peak in many parts of the world in winter because of increased heating, which is not a factor in the New Zealand summer.^12^, ^13^
3. Reporting effect: The Christmas holiday effect has now been described in at least 3 different regions,^1^, ^4^ including New Zealand. This would argue against a reporting effect being a major contributor to any apparent Christmas holiday effect. It would require an assumption that a common or comparable issue was present in all reporting and recording systems represented, which, although not impossible, is considered unlikely.
4. Month‐boundary effect: The Christmas holiday effect is not correlated with a monthly reporting cycle. It has also has been observed in a number of independent jurisdictions, reducing the likelihood of a month‐boundary effect.

Other suggested explanations are not clarified by the new results:

1. Changes in diet and alcohol consumption: The contribution of this factor cannot be further elaborated because it is a function of the holiday period and not of seasonality. Furthermore, the degree of overlap between the general holiday traditions and timing of events between New Zealand and the United States makes any distinction difficult without high‐quality dietary data. Assessing the impact of major holidays on cardiac mortality in cultures that have cultural attributes, including major holidays, distinct from New Zealand and Australia culture may aid in clarification of any dietary component.
2. Emotional stress associated with holidays: The contribution of this factor cannot be further elaborated because this is a function of the holiday and not of seasonality.
3. Inappropriate delay in seeking medical care: The Christmas holiday period is a common time for travel within New Zealand, with people frequently traveling away from the main centers. This could contribute to delays in seeking treatment because of both a lack of familiarity with nearby medical facilities and geographic isolation from appropriate medical care in emergency situations. The reduced age at death (Table 1) recorded during the Christmas period for persons with cardiac‐related mortality compared with both the non‐Christmas and pre‐Christmas periods (≈1 year and 6 months less, respectively) could add weight to the argument that this item is indeed a factor.
4. Displacement of death: This would include both hastening and postponement of death for reasons associated with the holiday period. The ability of persons to modify their date of death on the basis of dates of significance has been both confirmed^14^, ^15^ and refuted^16^, ^17^ elsewhere; however, it remains a possible explanation for this holiday effect. Because the Christmas effect is seen in cardiac deaths, this limits or eliminates the involvement of suicides, in which the time of death is much more directly controlled by the individual than other modes of mortality. The hastening or delaying of mortality would be expected to create a displacement effect in which a local peak is developed on or around dates of significance, leading to decreases in events surrounding the period in a compensatory manner.

The 2 explanations that are most supported by the data are displacement of death and inappropriate delay in seeking medical care. The modification of the age at death would appear to suggest that access to medical treatment, which would otherwise preserve life, is not available during this period. With regard to displacement of death, the trend within the data is toward concentration of cardiac mortality during the Christmas period, with lower than expected results (but not statistically significant) before and after Christmas.

Although the methods presented closely replicate those used in prior publications, some limitations of this current study could be addressed in future work. Ideally, daily temperature would have been linked to mortality events; however, this was not possible with this data set at this stage. This is a limitation, but a recent report indicated that the majority of mortality attributable to temperature was caused by cold conditions, with excessive heat playing a reduced role.^2^ This should mitigate the impact of not directly accounting for temperature variation in this data set, because the observed Christmas effect occurs during the summer season. New Zealand also has a temperate, island climate, which almost eliminates the extremes of temperature^18^ that have been associated with increased cardiovascular disease mortality rates.^19^, ^20^ The historical temperature records also indicate that the hottest period of the year in New Zealand typically falls outside of the identified Christmas periods.^21^

Because of the LOESS method used to estimate the “usual” event rate, it is likely that the effect of the Christmas period is underestimated due to the smoothing procedure incorporating part of the Christmas holiday effect into the estimate of expected events. This can be observed in Figure 2, in which a prominent deviation upward during the Christmas period can be observed on the smoothing or expected line (black line). The partial incorporation of the increase associated with the Christmas holiday effect would be expected to bias the result toward the null result of “no Christmas effect,” making it less likely to detect any true Christmas effect. A sensitivity analysis conducted to test this assumption indicated that the underestimate is on the order of 0.8%, or ≈20% of the detected effect. The result thus represents a conservative estimate, and there is confidence that, on the basis of the data available, the Christmas effect seen in the New Zealand population is of at least the magnitude reported. On this basis, and to maximize comparability with the paper by Philips et al,^1^ we maintained the LOESS method for this analysis.

In conclusion, we observed a clear Christmas holiday effect in the New Zealand population for persons suffering a cardiac death outside of a hospital setting, replicating the effect observed in US data. This result in the Southern Hemisphere, with seasonality opposite that in previous reports, helps reaffirm that there is no apparent correlation between any observed Christmas holiday effect and the impact of temperature or seasonality. We found that the age of death was younger for those who died from a cardiac‐related condition during the Christmas period compared with those who died of a similar condition during the remainder of the year and in the pre‐Christmas period. By virtue of studying data from a Southern Hemisphere location, we have been able to clarify the likely causes contributing to the Christmas holiday effect; however, we are not able to make a definitive statement about the cause of the effect. There is the possibility of a displacement effect, in which mortality is being concentrated during the holiday period rather than directly causing additional mortality; however, the use of a different method of estimating the expected deaths will be required to fully explore this issue.

## Sources of Funding

This research has been partially funded by 2 research bodies; an Australian National Health and Medical Research Council grant (NHMRC1084347) and a New Zealand Health Research Council grant (HRC49).

## Disclosures

None.

## Supporting information


**Table S1.** Percentage Difference Between Actual and Expected Mortality Event Counts for a Range of Potential Holiday Periods
**Table S2.** Percentage Difference Between Calculated Mean Mortality Count and Expected Cardiac Mortality Out of a Medical Facility Where the Christmas Effect Has Been Linearly Interpolated
**Table S3.** Daily Mean Values for Actual and Expected Mortality Events Divided by Event Type, Location, and Time Period
**Figure S1.** Cumulative frequency distributions of cardiac mortality in New Zealand 1988–2013.
**Figure S2.** Comparison of cumulative distribution function for cardiac mortality in the Christmas (blue line) and pre‐Christmas (red line) periods.
**Figure S3.** Mean number of cardiac mortality events occurring outside of a health facility between 1988 and 2013 (points) and locally weighted smoothing representing the expected values for the non‐Christmas period and a linear interpolation for the Christmas period. Each point represents a mean number of mortality events for a particular calendar day, with the color coding representing the time periods of significance with regard to the impact of the holiday effect.
**Figure S4.** Christmas effect stratified by time period.
