# Supplementary material for: Revisiting the “Christmas Holiday Effect” in the Southern Hemisphere
Source: J Am Heart Assoc. 2016 Dec 22;5(12):e005098. doi: 10.1161/JAHA.116.005098 (PMC5210403; doi:10.1161/JAHA.116.005098)

Supplemental Material

Table S1. Percentage difference between actual and expected mortality event counts for a range of potential holiday periods.

Christmas period dates		Christmas effect (%)	Lower CI	Upper CI
Start	end			
29th December	11th January	3.7	7.4	-0.009
28th December	10th January	4.1	7.9	0.3
27th December	9th January	4.5	7.9	1.1
26th December	8th January	4.3	7.8	0.9
25th December	7th January	4.2	7.7	0.7
24th December	6th January	3.8	7.4	0.2
23rd December	5th January	2.6	6.3	-1.2
22nd December	4th January	2.0	5.9	-1.9
21st December	3rd January	0.9	4.5	-2.7

Table S1 indicates that the timing of the Christmas effect is sensitive to the window of time over which it is calculated indicating that the effect is temporally specific.

Table S2. Percentage difference between calculated mean mortality count and expected cardiac mortality out of a medical facility where the Christmas effect has been linearly interpolated.

Time period	Percent difference between actual and expected	Upper confidence interval	Lower confidence interval
Christmas	5.03	8.58	1.45
Pre-Christmas	-0.34	3.02	-3.69
Post-Christmas	-3.03	0.62	-6.69
All non-Christmas	-0.08	0.55	-0.72

Table S3. Daily mean values for actual and expected mortality events divided by event type, location and time period.

Event Type	Location	Measure	Christmas	Pre-Christmas	Post-Christmas	All non-Christmas
Cardiac deaths	Not in a medical Facility	Actual	9.7	9.1	8.7	10.1
		Expected	9.3	9.1	9.0	10.1
	In a medical facility	Actual	9.3	9.7	9.0	10.7
		Expected	9.3	9.7	9.1	10.7
Non-cardiac deaths	Not in a medical Facility	Actual	19.2	18.5	18.3	19.4
		Expected	18.8	18.6	18.3	19.4
	In a medical facility	Actual	34.6	35.6	34.6	37.8
		Expected	34.7	35.2	34.4	37.8
All deaths	Not in a medical Facility	Actual	28.8	27.6	27.1	29.5
		Expected	28.0	27.7	27.2	29.5
	In a medical facility	Actual	43.8	45.3	43.6	48.5
		Expected	44.1	44.9	43.5	48.5

Figure S1. Cumulative frequency distributions of cardiac mortality in New Zealand 1988 - 2013

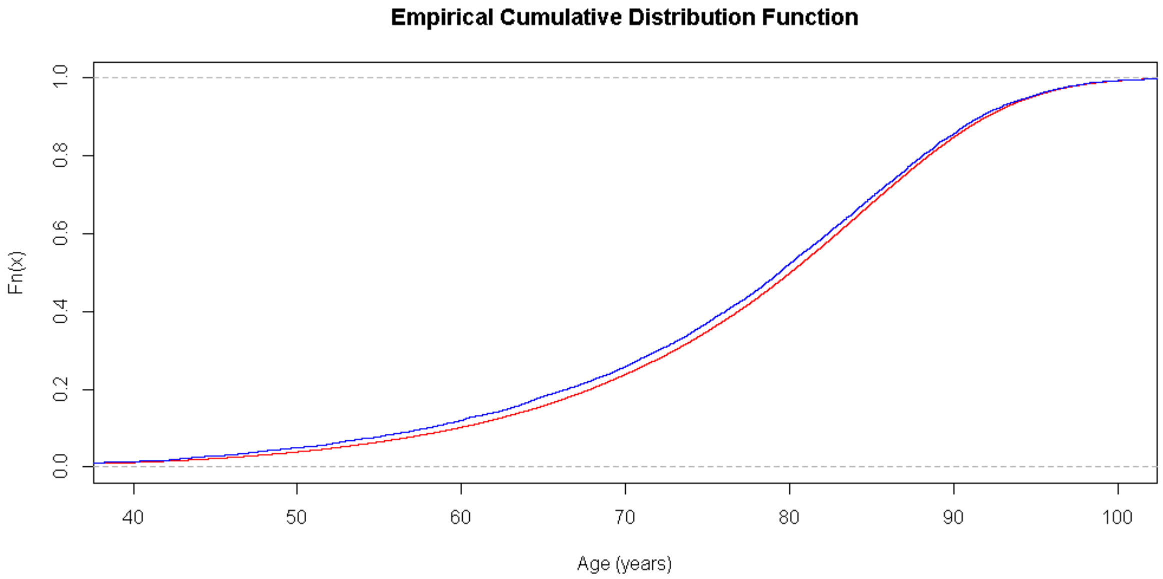

Comparision of cumulative distribution function for cardiac mortality in the Christmas (blue line) and non-Christmas (red line) periods.

Figure S2. Comparision of cumulative distribution function for cardiac mortality in the Christmas (blue line) and pre-Christmas (red line) periods.

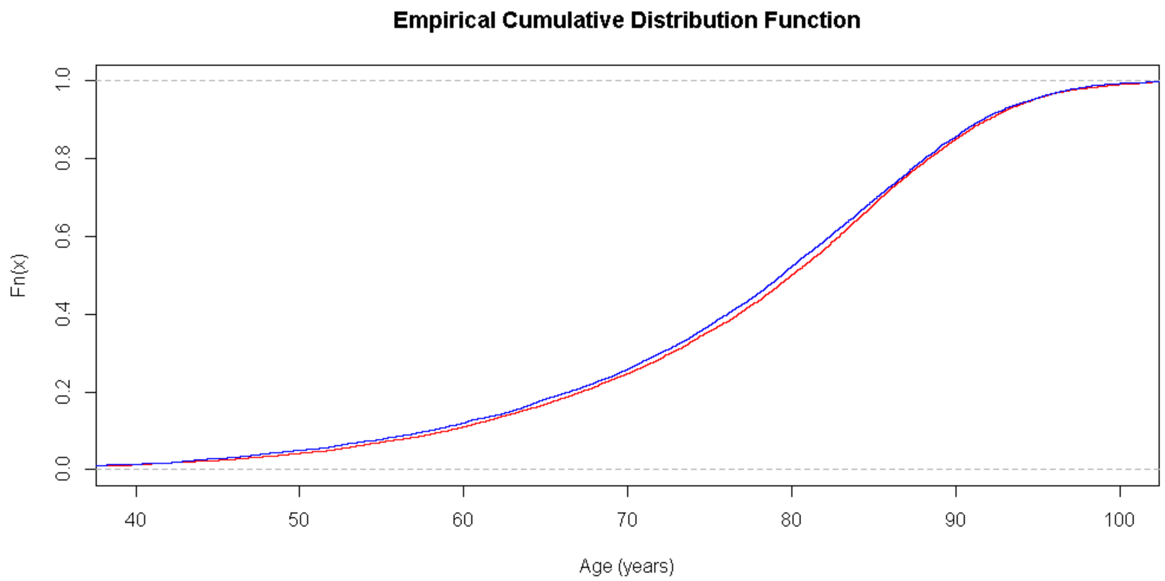

Figure S3. Mean number of cardiac mortality events occurring outside of a health facility between 1988 and 2013 (points) and a LOESS smooth representing the expected values for the non-Christmas period and a linear interpolation for the Christmas period. Each point represents a mean number of mortality events for a particular calendar day with the colour coding representing the time periods of significance with regard to the impact of the holiday effect.

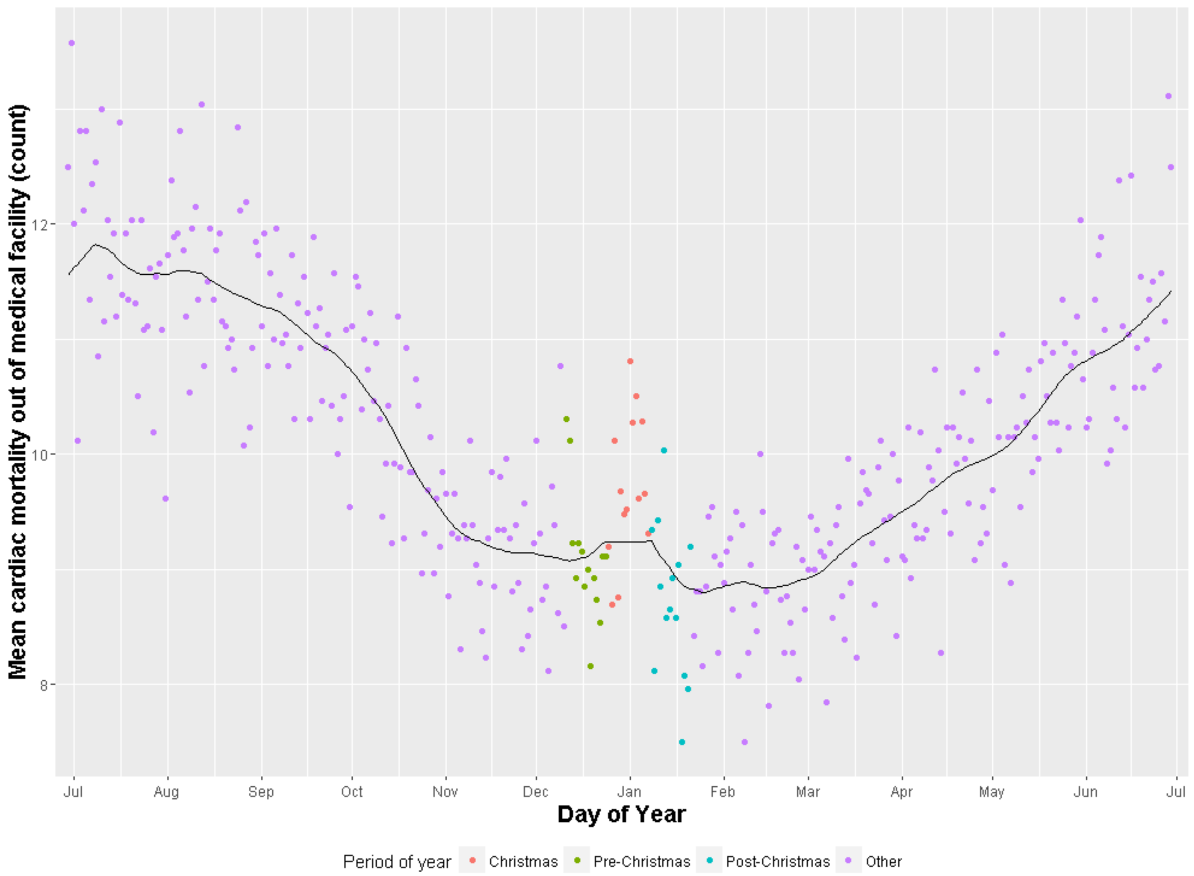

Figure S4. Christmas effect stratified by time period

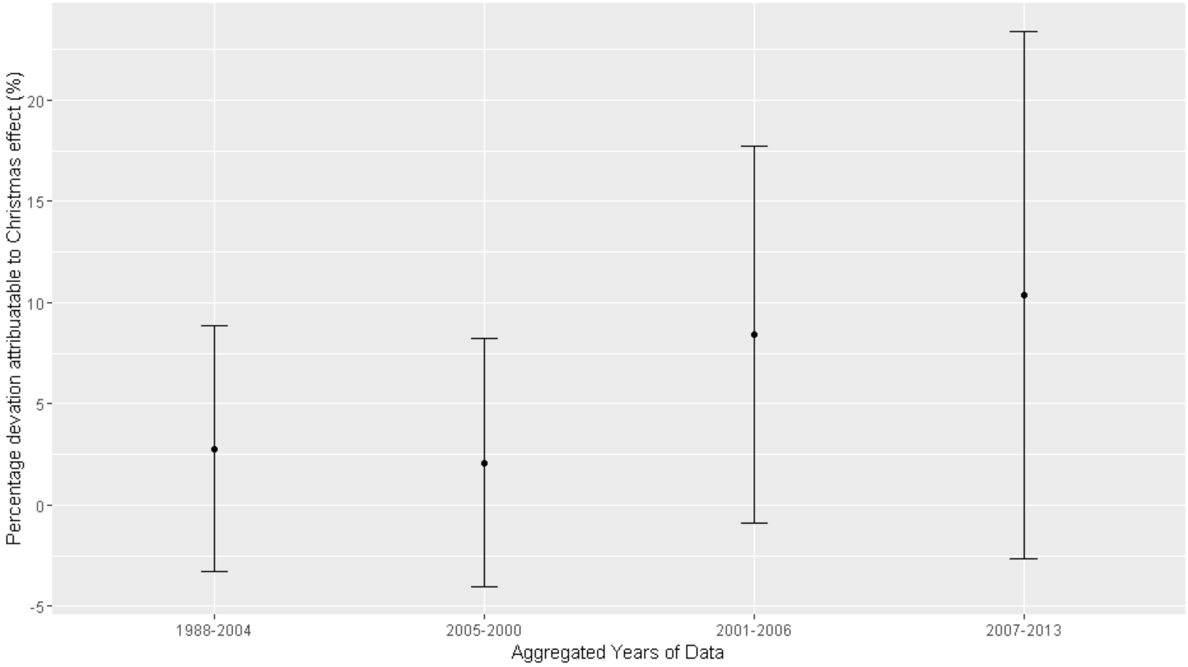

Supplement: Supplementary file 1 — Table S1. Percentage Difference Between Actual and Expected Mortality Event Counts for a Range of Potential Holiday Periods Table S2. Percentage Difference Between Calculated Mean Mortality Count and Expected Cardiac Mortality Out of a Medical Facility Where the Christmas Effect Has Been Linearly Interpolated Table S3. Daily Mean Values for Actual and Expected Mortality Events Divided by Event Type, Location, and Time Period Figure S1. Cumulative frequency distributions of cardiac mortality in New Zealand 1988–2013. Figure S2. Comparison of cumulative distribution function for cardiac mortality in the Christmas (blue line) and pre‐Christmas (red line) periods. Figure S3. Mean number of cardiac mortality events occurring outside of a health facility between 1988 and 2013 (points) and locally weighted smoothing representing the expected values for the non‐Christmas period and a linear interpolation for the Christmas period. Each point represents a mean number of mortality events for a particular calendar day, with the color coding representing the time periods of significance with regard to the impact of the holiday effect. Figure S4. Christmas effect stratified by time period. [file JAH3-5-e005098-s001.pdf]
